# Salivary Microbiological and Gingival Health Status Evaluation of Adolescents With Overweight and Obesity: A Cluster Analysis

**DOI:** 10.3389/fped.2020.00429

**Published:** 2020-07-31

**Authors:** Darlle Santos Araujo, Marlise Inêz Klein, Kelly Guedes de Oliveira Scudine, Luana de Sales Leite, Thais M. Parisotto, Caroline Marcantonio Ferreira, Fernando Luiz Affonso Fonseca, Matheus Moreira Perez, Paula Midori Castelo

**Affiliations:** ^1^Department of Pediatric Dentistry, Universidade Estadual de Campinas (UNICAMP), Piracicaba, Brazil; ^2^Department of Dental Materials and Prosthodontics, São Paulo State University (UNESP), School of Dentistry, Araraquara, Brazil; ^3^Laboratory of Molecular Biology of Microorganisms, São Francisco University, Bragança Paulista, Brazil; ^4^Department of Pharmaceutical Sciences, Universidade Federal de São Paulo (UNIFESP), Diadema, Brazil; ^5^Department of Pathology, ABC Medical School, Santo André, Brazil

**Keywords:** obesity, saliva, microbiota, oral health, adolescent

## Abstract

Given the high prevalence of obesity in children and adolescents, the investigation of early markers is of clinical importance to better manage this condition. Thus, the aim was to evaluate the cross-sectional relationship between salivary microbiota, gingival health status, and excess weight in adolescents. A total of 248 students (14–17 y; 119 girls) were included, free of caries lesions and periodontal pockets. Physical examination included measures of height, weight, and body fat percentage (%BF). Oral examination was performed to gather information on dental (DMFT index) and gingival health status. Unstimulated saliva was submitted to qPCR reactions to quantify *Streptococcus mutans, Porphyromonas gingivalis*, Bifidobacteria, and *Streptococcus pneumoniae* percentages and the NFKappaB expression. Two-way ANOVA was applied considering *group* (normal-weight/overweight/obesity) and *sex* factors, in addition to cluster analysis. *Group* effect was significant for %*S. mutans* (partial eta^2^ = 0.20; *p* < 0.001) and %Bifidobacteria (partial eta^2^ = 0.19; *p* < 0.001), with overweight and obesity groups showing the highest levels compared to normal-weight ones, with no significant *sex* effect. There was no difference in the frequency of gingivitis, *P. gingivalis*, and *S. pneumoniae* percentages or NFKappaB expression between groups. Cluster analysis generated three clusters according to body fat accumulation: “Higher %BF,” “Moderate %BF,” and “Lower %BF.” “Higher %BF” cluster was characterized by higher body fat percentage and higher salivary %Bifidobacteria, while cluster “Lower %BF” was characterized by lower body fat percentage and lower frequency of gingivitis (“Moderate %BF” cluster was the contrast). According to nutritional status, a difference in salivary *S. mutans* and Bifidobacteria percentages was found, with overweight or obesity adolescents showing the highest percentages than normal-weight ones. Besides, a positive relationship between body fat accumulation and Bifidobacteria count was observed, indicating a possible interaction between oral bacteria communities and weight gain.

## Introduction

Obesity is a prevalent condition associated with a chronic low-grade inflammation that predisposes insulin resistance and the development of type-2 diabetes mellitus. In 2016, over 340 million children aged 5–19 years were overweight or had obesity (1). Although its prevalence among high-socioeconomic status adolescents has decreased over the years, the prevalence of obesity among low-socioeconomic status has continued to increase (2). Therefore, it is paramount to find early markers for obesity to better manage this condition.

Diet can interfere with the oral (3, 4) and gut microbiome (5, 6) and this, in turn, influences the immune system and the development of obesity. Changes in the gut microbiota of individuals with overweight have been reported (6–10). However, not only the gut microbiota is associated with diseases, but changes in the oral microbiota have also been related to systemic diseases (11–13); as the oral cavity is part of the mucosal system they may thus influence the immune system and homeostasis. The oral cavity has the second most diverse microbial community in the body (14), and it is characterized as the gateway for microorganisms that are swallowed daily together with saliva (14, 15), acting as an inoculum to the gut in which the microorganisms that find adequate conditions to colonize can give rise to distinct types of gut communities.

The analysis of salivary cytokines levels (16) and oral microbiome (17, 18) may be useful for better understanding and monitoring chronic diseases such as obesity. It was reported that Bifidobacteria levels in the mouth seem to reflect those of the lower gastrointestinal tract (19) and, to our knowledge, there is a lack of studies that evaluated bacterial species related to oral and systemic diseases in the saliva of a large sample of adolescents with excess weight.

In this sense, the study hypothesized that saliva might exhibit different profiles and quantities of inflammatory markers and specific bacterial species/genera in the presence of excess weight in adolescence, which may ultimately predispose the individual to both oral and/or systemic diseases. Thus, the aim was to examine the relationship between salivary microbiota, gingival health status, and excess weight in a large sample of adolescents, also identifying homogenous groups of individuals according to body fat accumulation, salivary microbiota, and inflammatory marker.

## Materials and Methods

### Participants

This work is a cross-sectional analytical study approved by the Research Ethics Committee of the Piracicaba School of Dentistry (University of Campinas, Brazil; protocol no. 152/2014), which followed all the principles of the Declaration of Helsinki. A convenience sample of 248 consented adolescents were included, who were selected from 12 public schools of the municipality of Piracicaba (urban area, countryside of the State of São Paulo, southeast, Brazil) in the year of 2016 and participate in all stages of data collection. The signed Assent Form and the Informed Consent Form were gathered from all of them and their parents, respectively. All physical and clinical examinations, including the interview, were carried out on the same day; the total sample of adolescents (248) participated in all stages of the study.

The sample size was calculated based on the results of Zeigler et al. (20), which evaluated the subgingival biofilm bacterial count in adolescents with obesity using a statistical package (BioEstat 5.0; Mamirauá, Belém, PA, Brazil). Given the 80% test power, and an alpha level of 0.05, a minimum of 200 subjects should be included in the study. For this reason, a 20% larger sample was included to compensate for losses.

### Interview

An interview was performed to gather information on dental and medical history and medication use profile, which was performed by a trained examiner (DSA). Female participants were asked about the date of menarche and the use and type of contraceptive medication (15, 21, 22). The inclusion factors were adolescents of both sexes and girls who have reached menarche.

The exclusion factors considered were: dental caries lesions (cavities), tooth loss, and periodontal disease (periodontal pockets >3 mm); chronic diseases such as epilepsy, cancer, rheumatoid arthritis, asthma, hypertension, or diabetes mellitus; chronic use of medications such as benzodiazepines, anti-inflammatories, corticosteroids, antidepressants, and contraceptives; use of tobacco or illicit drugs; antibiotic use in the last 3 months (23); use of xerogenic medications and/or symptoms of xerostomia; refusal to collaborate with the proposed activities.

### Oral Examination

The oral examination was performed at schools with daylight (but not direct sunlight) using mirror, probe, and personal protective equipment; the presence of caries experience was examined using the DMFT index (total decayed, missed, and filled teeth) (24) by a calibrated examiner (DSA, Public Health Spec.; Kappa coefficient = 0.97). The inclusion criterion was the presence of complete permanent dentition, except for the third molars, free of active caries lesions (free of cavities).

The gingivitis diagnosis was performed by the same examiner (DSA), based on subjective and objective measures: the self-perception of gingivitis and the presence of gingival bleeding on probing, which was examined using the Community Periodontal Index (IPC) and WHO probe (24). The self-perception of gingivitis was based on an interview considering two questions with dichotomous answers (yes/no): current perception of gingival bleeding and gingival bleeding during toothbrush. Those questions were based on previous studies performed in children and adolecents (25, 26). Thus, the presence of gingivitis was considered positive in the presence of symptoms and bleeding on probing in at least one sextant (27).

### Physical Examination

Anthropometric and nutritional assessments involved measurements of height, weight, body fat mass, skeletal muscle mass, and intracellular and extracellular water using a digital stadiometer and bioelectrical impedance analysis (InBody 230, Biospace Co. Ltd., Seoul, South Korea). All examinations were performed at schools, by a trained examiner (DSA) in a private room.

Body mass index (BMI = Kg/m^2^) was used to classify the sample into normal-weight, overweight, and obesity as described earlier (28, 29).

### Saliva Collection

Unstimulated saliva collection was performed at schools between 8:00 and 10:00 h, at least 2 h after the last meal, and 1 h after oral hygiene in order to avoid circadian variations. The participant, seated comfortably in a chair, with her/his head slightly tilted forward without swallowing, allowed the saliva accumulated on the floor of the mouth for 5 min to drip into a previously weighed tube dipped in crushed ice. The samples were transported on ice to the laboratory on the same day and stored at −40°C until analysis.

Females were evaluated in the most stable week of the menstrual cycle, i.e., the follicular phase (3rd to 5th day of the cycle), to control variations in the menstrual cycle. All volunteers were instructed to avoid alcohol consumption and physical exercise on the day before the collection.

### Microbiological Investigation

The microbial DNA in unstimulated saliva samples was isolated and subjected to quantitative PCR reactions (qPCR) to quantify the microorganisms, as previously described (21). The specific primers used were for target microorganisms *Streptococcus mutans* (30), *Porphyromonas gingivalis* (31), *Streptococcus pneumoniae* (32), Bifidobacteria (33), and total bacteria (34). For the isolation of genomic DNA, 1 ml of saliva was centrifuged (13,000 xg/10 min/4°C) and the supernatant discarded. The precipitate was suspended in 500 μl of TE buffer (50 mM Tris, 10 mM EDTA, pH 8.0) and 1.5 μl of propidium monoazide (20 mM in 20% dimethylsulfoxide; Biotium, Hayward, CA) (35). After incubation for 5 min in the dark, under agitation, the samples were exposed to light (600 W) for 3 min. Following the light-induced crosslinking to extracellular DNA, samples were centrifuged (5,000 g/5 min/4°C), and the supernatant discarded. The precipitate was then suspended in 100 μl of TE buffer, 10.9 μl lysozyme (stock 100 mg/mL, Sigma-Aldrich, USA) and 5 μl mutanolysin (stock 5 U/μL, Sigma-Aldrich, USA). This suspension was incubated at 37°C for 30 min. Next, DNA was isolated with the MasterPure DNA Purification kit (Epicenter Technologies, Madison, Wis, USA) following the manufacturer's recommendations. The amount and purity of the DNA were assessed by OD260 nm and the ratio OD260/280, respectively. The template for microbial quantitation was 10 pg and 100 ng of genomic DNA from each sample (based on the detection of specific species in the qPCR reaction) and the negative controls (no DNA). These templates were mixed with Bimake 2x SYBR Green qPCR Master Mix (Biotool, Bimake, TX, USA) and species-specific primers; the concentrations of each primer and their respective annealing temperature and qPCR cycle conditions were as previously published (30–34). The reactions were carried out with a CFX96 system (Bio-Rad Laboratories, Inc., Ca, USA).

The standard curves were based on the genome size of each microorganism and average of total oral bacteria available genomes (2.45 Mb), according to Dolezel et al. (36). Specifically, a copy of the genome is a cell of each organism. The curve was used to transform the values of the critical threshold cycle (Ct) to cell number. Standard curves were constructed using genomic DNA from standard ATCC (American Type Culture Collection) strains. Data were presented as a proportion of each species to the total bacterial load, which was determined using 16S rRNA universal primer.

### NFKappaB Expression

One mL of saliva with a preservative solution (RNAlater, Ambion) was transferred to a conical tube, and RNA isolation was performed with Trizol® Reagent according to the manufacturer's recommendations. RNA quantity and purity were measured by spectrophotometry. Samples with a ratio of 260/230 and 260/280 between 1.8 and 2.0 were considered valid. cDNA was synthesized with the QuantiNova Reverse Transcription Kit (Qiagen) from 1 μg total RNA and according to the manufacturer's instructions. Gene expression was evaluated by RT-qPCR using SYBR Green.

Primers were made using the Primer-Blast platform available at http://www.ncbi.nlm.nih.gov/tools/primer-blast/. The sequence of the primer is described below. The following reaction conditions for RT-qPCR were used: 7.5 μl SYBR Green PCR Master Mix (SABioscience), 0.3 μl forward and reverse primers (0.25 uM), 5.9 μl water and 1 μl cDNA, subject to the following cycling condition: 95°C, 10 min; 40 cycles of (95°C, 15 s; 60°C, 60 s), using the Applied Biosystem 7500. The reference gene used for normalization of NF-Kappa B expression values was β-Actin (NF-KB Forward: CATCCCATGGTGGACTACCT; NF-KB Reverse: CTCTGTCATTCGTGCTTCCA [Pb100]; β-actin Forward: CCCTGGAGGAGAGAGAG; β-actin Reverse: CAATGCCAGGGTACATGGTG [Pb100]).

### Statistical Analysis

Data were analyzed using SPSS 24.0 software (IBM Corp., NY, USA) by one of the authors (PMC, Applied Statistics Spec), considering an alpha level of 5%. Exploratory statistics consisted of means, standard deviation, medians, and quartiles. Normality was tested by the Kolmogorov-Smirnov test and QQ-plot graphs; variables that did not present normal distribution were transformed by the natural logarithm (Ln). Missing values occurred due to salivary analysis, although we opted not to perform any kind of data imputation.

The evaluation of oral health parameters comprised the comparison of gingival status between groups using the Chi-square test. In contrast, the DMFT index and the number of sextants with gingival bleeding on probing were compared between groups by Kruskal-Wallis test, considering the sexes separately.

Two-way ANOVA (and Bonferroni post-test) was used to verify the *group* (normal-weight, overweight, or obesity) and *sex* (male and female) effect and the interaction between these factors on the variance observed in %*S. mutans*, %*P. gingivalis*, %Bifidobacteria, and %*S. pneumoniae*, and NFKappaB expression in saliva. Effect size (partial Eta squared) and the power of the test were also used for interpretation. Previously, the results of the homogeneity test (Levene's test) were evaluated as a premise of variance analysis. A Bonferroni-type adjustment was performed to prevent alpha inflation; thus, the alpha level was adjusted to 0.01.

Data were also submitted to cluster analysis to identify homogenous groups of adolescents according to body fat accumulation. First, hierarchical cluster analysis using the farthest neighbor method for calculating distances between clusters was performed to obtain the dendrogram and analyze the range of clusters for further running K-means (37). K-means analysis was performed to identify homogenous groups of adolescents, according to %BF, *%S. mutans*, %Bifidobacteria, *%P. gingivalis, %S. pneumoniae*, expression of NFKappaB, and the frequency of gingivitis. The pairwise deletion procedure was chosen because of the missing data related to NFκB measures. The final number of clusters was based on the interpretability and reliability of the cluster solution; the silhouette coefficient was considered for internal clustering validation. For descriptive purposes, differences between clusters were assessed by observing the *F*-value.

## Results

Table 1 shows the anthropometric and clinical characteristics of the sample. There was no difference in caries experience (male: *p* = 0.536; female: *p* = 0.124), frequency of gingival bleeding (males: *p* = 0.110; females: *p* = 0.702), and in the frequency of gingivitis (males: *p* = 0.078; females: *p* = 0.495) between groups classified according to BMI. Additionally, all three groups were homogeneous for age.

**Table 1:** Anthropometric and clinical characteristics of the adolescents.

**Group**	***n***	**Age (y)**	**BMI (Kg/m^2^)**	**%Body fat**	**DMFT index**	**Spontaneous gingival bleeding**	**Gingival bleeding when brushing teeth**	**Gingivitis (self-report and clinical)**	**Number of sextants with gingival bleeding**
		**Mean (SD)**	**Mean (SD)**	**Mean (SD)**	**Median (25–75%)**	**%**	**%**	**%**	**Median (25–75%)**
Girls normal-weight	66	15.7 (0.9)	20.2 (2.2)	26.2 (5.5)	0 (0–2)	15.4	32.3	18.5	0 (0–1)
Boys normal-weight	76	15.5 (0.7)	20.2 (2.2)	14.0 (5.7)	0 (0–3)	2.6	27.6	9.3	0 (0–1)
Girls overweight	30	16.1 (0.5)	26.3 (2.0)	38.3 (4.2)	1.5 (0–6)	13.8	51.7	24.1	0.5 (0–2)
Boys overweight	32	15.1 (0.5)	25.6 (1.4)	23.4 (4.9)	1 (0–2)	6.3	43.8	15. 6	0 (0–1)
Girls obesity	23	16.3 (1.0)	33.5 (4.5)	45.8 (3.7)	1 (0–2)	16.0	48.0	20.0	0 (0–2)
Boys obesity	21	15.7 (1.1)	31.3 (3.5)	33.1 (5.3)	0 (0–1)	14.3	42.9	28.6	1 (0–1)

*BMI, body mass index; DMFT, decay-missing-filled index*.

**Figure 1:**
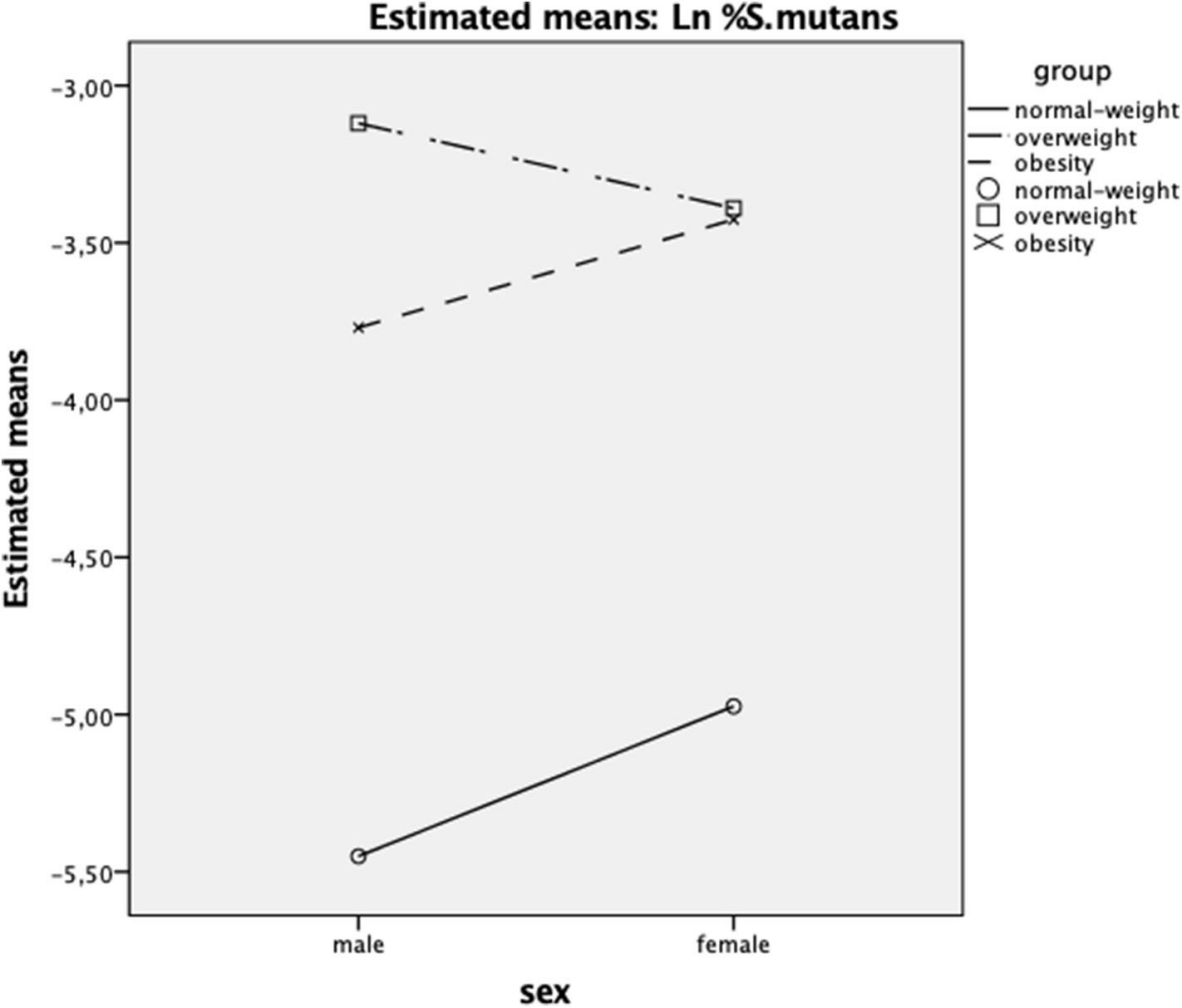
Interaction between *sex* (women/men) and *group* factors (normal-weight/overweight/obesity) on %*S. mutans* (Y axis). Ln, natural logarithm transformation.

Table 2 summarizes the results found for salivary microbiological analysis of the groups, as well as the results obtained in the evaluation of NFKappaB expression in saliva. The exact number of observations are expressed in Tables 1, 2. A significant *group* effect was found for %*S. mutans*; Bonferroni post-test showed that the normal-weight group showed the lowest percentage, with a large effect size and a power >80% (*p* < 0.001) (Figure 1). The *sex* factor had no effect on %*S. mutans* and no significant interaction group^*^sex was observed.

**Table 2:** Percentage of *S. mutans, P. gingivalis*, Bifidobacteria, and *S. pneumoniae* in relation to total bacterial load and expression of inflammatory marker NF Kappa B in saliva: a two-way general linear model (Median; 25–75%).

**Group**	**% *S. mutans***		**% *P. gingivalis***		**% Bifidobacteria**		**% *S. pneumoniae***		**NF-KB 2^−^ΔCt**	
Girls normal-weight	471E-05 (128E-05–391E-05)		2E-05 (<1E-05–29E-05)		649E-05 (110E-05–4550E-05)		5E-05 (<1E-05–44E-05)		0.0022 (0.0014–0.0107)	
*n*	66		66		66		66		18	
Boys normal-weight	376E-05 (75E-05–139E-05)		2E-05 (<1E-05–16E-05)		208E-05 (32E-05–735E-05)		3E-05 (<1E-05–27E-05)		0.0038 (0.0021–0.0080)	
*n*	76		76		76		76		29	
Girls overweight	743E-05 (222E-05–413E-05)		1E-05 (<1E-05–9E-05)		908E-05 (119E-05–2960E-05)		2E-05 (<1E-05–23E-05)		0.0006 (0.0004-0.0067)	
*n*	30		30		30		30		5	
Boys overweight	484E-05 (51E-05–238E-05)		<1E-05 (<1E-05–5E-05)		200E-05 (49E-05–1510E-05)		1E-05 (<1E-05–10E-05)		0.0014 (0.0005–0.0028)	
*n*	32		32		32		32		8	
Girls obesity	140E-05 (177E-05–461E-05)		12E-05 (1E-05–163E-05)		1000E-05 (178E-05–2870E-05)		4E-05 (1E-05–7E-05)		0.0011 (0.0002–0.0020)	
*n*	23		23		23		23		8	
Boys obesity	484E-05 (49E-05 – 131E-05)		1E-05 (<1E-05–35E-05)		388E-05 (58E-05–5540E-05)		7E-05 (<1E-05–176E-05)		0.0011 (0.0008–0.0053)	
*n*	21		21		21		21		6	
**Effect**	***p*-value**	**Eta partial^2^/power**	***p*-value**	**Eta partial^2^/power**	***p*-value**	**Eta partial^2^/power**	***p*-value**	**Eta partial^2^/power**	***p*-value**	**Eta partial^2^/power**
Group	**<0.001**	**0.198/1.00**	0.608	0.005/0.13	**<0.001**	**0.185/1.00**	0.535	0.007/0.15	0.819	0.006/0.08
Sex	0.609	0.002/0.08	0.843	<0.001/0.05	0.334	0.006/0.16	0.412	0.004/0.13	0.713	0.002/0.07
Group*sex	0.583	0.007/0.14	0.229	0.016/0.31	0.329	0.015/0.24	0.113	0.024/0.45	0.825	0.006/0.08

**Figure 2:**
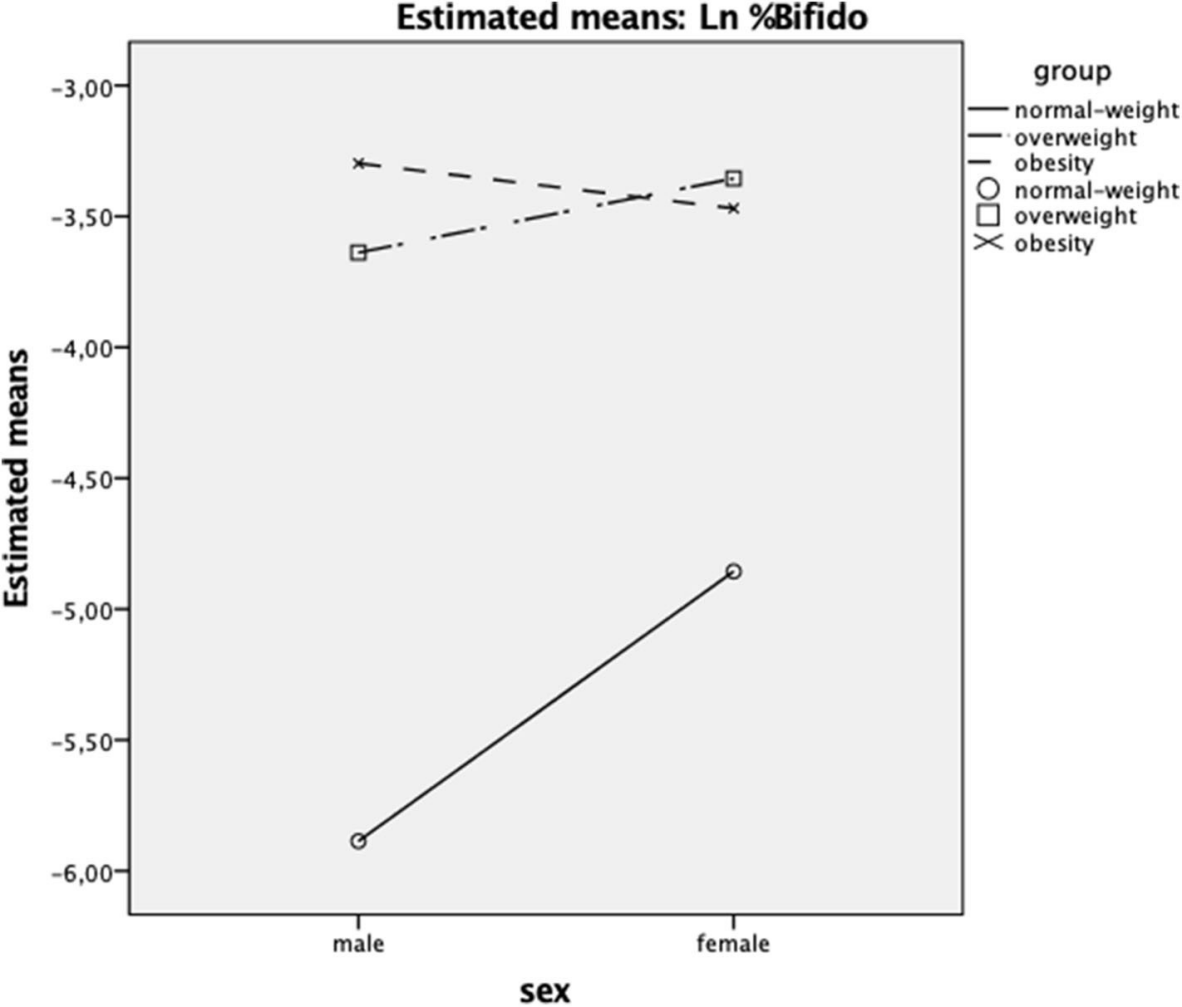
Interaction between *sex* (women/men) and *group* factors (normal-weight/overweight/obesity) on %Bifidobacteria (Y axis). Ln, natural logarithm transformation.

A significant *group* effect was found for %Bifidobacteria with a large effect size and a test power >80%; Bonferroni's post-test found differences between normal-weight, overweight, and obesity groups, with the lowest mean of %Bifidobacteria for the normal-weight group (Figure 2). The *sex* factor was not significant, neither the interaction group^*^sex.

In contrast, %*P. gingivalis*, %*S. pneumoniae*, and measures of NF-KB 2^Δ^Ct in saliva did not vary according to *group* or *sex*. However, it is important to mention the high percentage of missing data for NF-KB 2^Δ^Ct measurement, which may have weakened the comparison across groups, as observed by the low power of the test found (~8%; Table 2).

**Figure 3:**
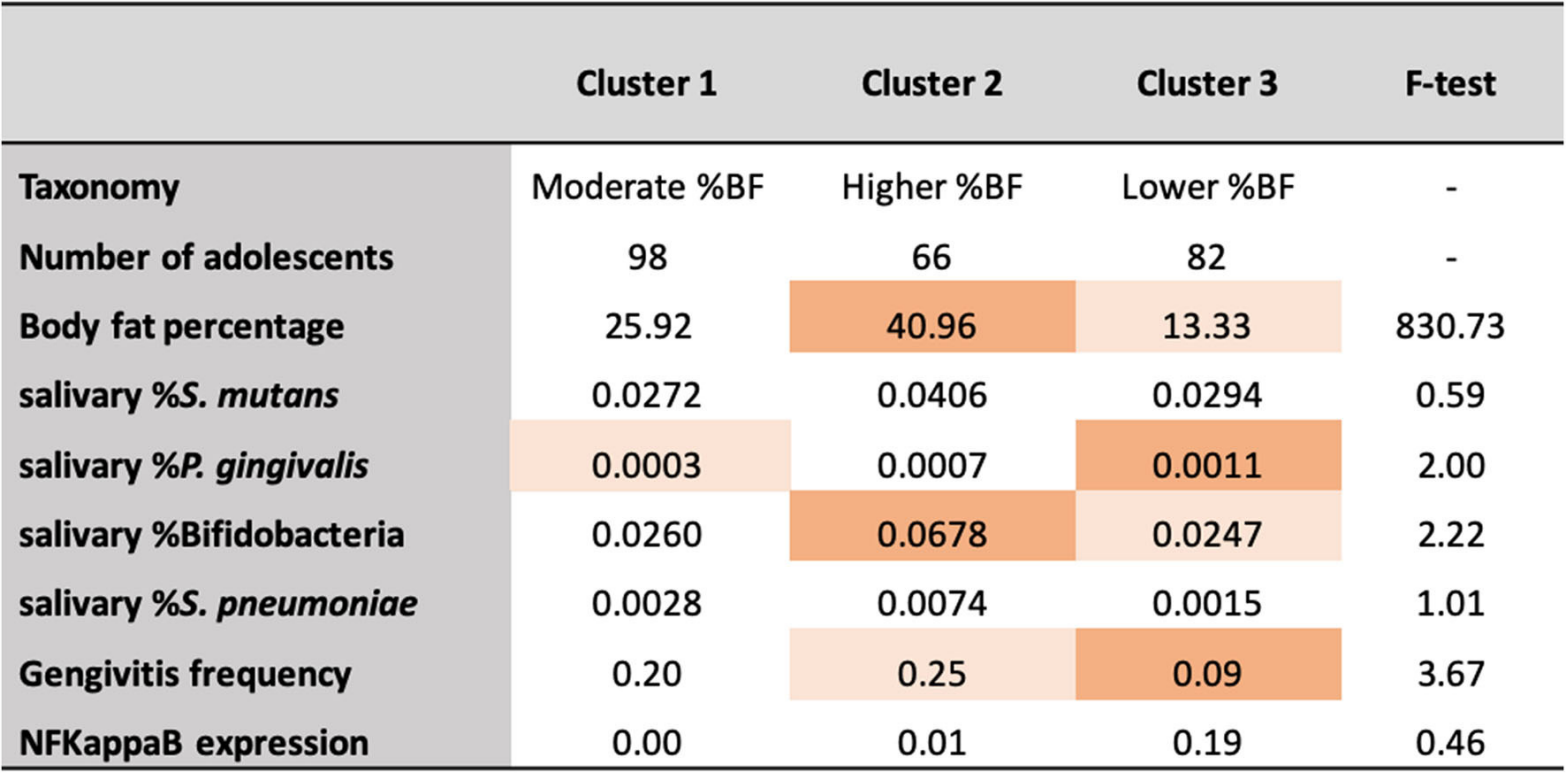
Final cluster centres (means) of the seven variables (*n* = 246) (%BF, Body fat percentage).

Cluster analysis was carried out considering the %BF measured by bioelectric impedance and identified three reliable and meaningful clusters (Figure 3), with an average silhouette width of 0.6 (acceptable internal validity). By interpreting the *F*-values above 2.0, the three clusters varied by %BF, salivary %Bifidobacteria, and the presence of gingivitis. For taxonomy purposes, Cluster 2 “Higher %BF,” which was characterized by higher %BF and higher salivary %Bifidobacteria, while Cluster 3 “Lower %BF” was characterized by lower %BF and lower frequency of gingivitis (Cluster 1 was the contrast). Of note, *F*-tests should be interpreted only for descriptive purposes (not considering alpha level), as the clusters were chosen to maximize the differences between each case and the other clusters. Thus, when considering the measures of %BF, the results of the cluster analysis emphasized the relation between body fat accumulation and %Bifidobacteria, while lower %BF was related to a reduced frequency of gingivitis.

## Discussion

The main findings of this study were the differences found in %*S. mutans* and %Bifidobacteria in the adolescents' saliva with different nutritional status. Adolescents with overweight or obesity showed the highest percentages of *S. mutans* and Bifidobacteria than normal-weight ones. Additionally, a cluster analysis emphasized the relationship between body fat accumulation and %Bifidobacteria, while lower %BF was related to a reduced frequency of gingivitis.

Salivary biomolecules can provide information on many organs and systems. Studies have pointed out that some types of bacteria found in saliva may reflect the individual's health status, including obesity (5, 38). However, studies that evaluated the impact of overweight independently of its comorbidities or other chronic diseases on adolescent's oral health and salivary microbiota are scarce although of importance since this is a particular age in which preventive measures can be introduced to improve health in the adulthood. Despite differences in the composition of the gut and oral microbiota, the types of communities observed in the gut appear to be predictive of the communities observed in the mouth, and vice versa (39). Oral inoculation with *P. gingivalis* in experimental models leads to changes in gut microbiota, which is a possible mechanism for the establishment of diseases associated with periodontitis, such as cardiovascular diseases (40).

In a previous sequencing study in Finnish children's salivary samples, Firmicutes (51%), Bacteroidetes (20%), Proteobacteria (16%), Actinobacteria (6%), Candidate division TM7 (4%), and Fusobacteria (3%) were the six major phyla found, (15) while Bifidobacteria did not seem to be highly prevalent in the Finnish children's samples. As the present results highlighted the relation between obesity and elevated salivary %Bifidobacteria levels in Brazilian adolescents, it may potentially represent a marker to identify those adolescents at increased risk of weight gain.

Recent studies have claimed that gut microbiota is a critical regulator of body weight (41), and *Bifidobacterium* species would improve metabolic status. In this sense, probiotics composed of *Lactobacillus* and *Bifidobacterium* have been used to modulate the gut microbiota, improving the intestinal immune barrier, and reducing the production of proinflammatory cytokines (42). Although no specific gastrointestinal pathogens were found to induce obesity or diabetes in experimental models, these conditions were associated with reduced intestinal Bifidobacteria abundance in previous studies (43, 44).

In patients undergoing gastroplasty, one study observed that before surgery patients with morbid obesity and high levels of hemoglobin A1c showed low levels of Bifidobacteria in saliva and feces, and 2 weeks after the surgery the levels of oral Bifidobacteria increased 10-fold while TNFα levels decreased, (19) although it is important to consider the influence of comorbidities involved in this relation. A Brazilian study involving children aged 3–11 years also found a negative correlation between BMI and Bifidobacterium spp. copy number in feces (45).

Although these studies suggested that Bifidobacteria are associated with weight control and beneficial aspects, some studies reported an association with other diseases. In fecal samples of ashmatic subjects, there was an increased prevalence of *Bifidobacterium adolescentis*, while in healthy individuals, the predominant species were *Bifidobacterium longum, Bifidobacterium brevi*, and *Bifidobacterium bifidum* (46). Interestingly, a study evaluating the effects of four Bifidobacteria on energy metabolism in rats showed that the effects are strain-dependent, and different responses, with gain or loss of weight, can be observed (47). Thus, future studies are needed to understand which Bifidobacterium species would represent a valid salivary marker of excess weight/weight gain.

There is evidence that Bifidobacterium species could use vitamin K, an essential factor for *P. gingivalis*, and the competition for this substrate would reduce the pathogen population (48). It is of note that Cluster 3 (called “lower %BF”) exhibited the higher %*P. gingivalis* and the lower %Bifidobacteria, agreeing with this possible interaction. The composition of the microbiota is considered one of the main factors in the development of one disease, and interspecies signaling in microbial communities could influence the nutrient acquisition, antagonistic substance production, and the expression of colonization and virulence factors (49). Given the complex relationship between bacterial species and variations on different compartments of the body, it is crucial to deepen the understanding of this interaction, especially in a multifactorial condition such as obesity.

Only adolescents without caries lesions and periodontal pockets were included, and this is probably why the %*P. gingivalis* did not differ between groups. However, adolescents with excess weight but free of dental caries showed the highest %*S. mutans* in saliva compared to normal-weight ones if considering that it is an important etiological factor for tooth decay and has been shown to decrease its levels after tooth restoration and caries lesion sealing (21). Indeed, oral bacterial diseases are not caused by single species but by a consortium of species in the oral cavity (50). A disease occurs when the microbiota associated with health is challenged with factors that trigger dysbiosis, following the concepts of the ecological plaque hypothesis (51). The current consensus is that dental caries is a sugar- and biofilm-dependent disease, once sugars start the process and trigger a causal chain (52). Specifically, the presence of dietary sugars in the mouth causes an ecological shift in the microbiota in the dental biofilm because it provides the substrate for acidogenic (saccharolytic) species to produce organic acids. These acids lead to acidic microniches within the biofilm and at the interface of teeth/biofilms that promotes the selection of aciduric species, and the demise of acid-sensitive species, over time, causes teeth demineralization that can evolve to cavities and eventual tooth loss if not treated (53). *S. mutans* is among the acidogenic and aciduric species that plays a role in the build-up of sturdier biofilms (53). In this context, previous studies showed that sucrose consumed several times a day in small quantities could induce changes in the salivary microbiome and *S. mutans* levels (3, 23) which may, ultimately, predispose these individuals to dental caries; others have also associated sugary food intake and BMI with differences in cariogenic microorganism counts (4, 54, 55).

Although the frequency of gingivitis did not differ between adolescents with different nutritional status, cluster analysis highlighted a relationship between lower body fat accumulation and a reduced frequency of gingivitis. According to some authors, the evidence supporting the existence of a common biological mechanism—inflammation—between fat accumulation and periodontal disease is not fully understood (56, 57) and it has been suggested the existence of a mediator in this process: the lifestyle, which is related to the concern with oral health (hygiene habits) and the amount and frequency of carbohydrate consumption (58). In children, significant altered levels of salivary inflammatory markers were found between individuals with normal-weight and excess weight (16, 59), while the gingival status was only affected when type 2 diabetes was present in children with obesity (22). In adolescents with obesity, the presence of periodontal pockets was associated with higher levels of IL-6 and raised diastolic blood pressure (60). In the present study, the analysis of NFKappaB expression in saliva could serve as a marker of subclinical inflammation; however, the analysis did not perform well and could not be quantified in many adolescents, as verified by the high number of missing values.

Oral bacteria have been linked to bacterial pneumonia in the elderly (61), and several anaerobic oral bacterial species may predispose to this disease (56, 62). Moreover, obesity has been associated to a greater susceptibility to respiratory infections, as studies suggested that leptin regulation, which is related to appetite and body weight, may exert a specific effect on the gut microbiota composition (63), specifically on *Klebsiella pneumoniae* and *S. pneumoniae* (64). Although the levels of *S. pneumoniae* did not differ between adolescents with different nutritional status, the investigation of whether hormones interact with oral and gut bacterial communities predisposing the individuals to systemic diseases and weight gain is of importance.

The evaluation of dietary habits in this large population was not feasible given the number of 24 h Recall questionnaire left incomplete or unanswered, which may be considered one of the limitations of the study. However, it is important to emphasize the large sample size included, composed of adolescents (students) free of comorbidities, which ensured statistical power to detect the large differences observed, and the control of potential confounding factors such as clinical oral status and medication intake (especially contraceptives). Further studies are needed to understand the role of oral bacteria communities in weight gain. Thus, the next steps should comprise a broader evaluation of salivary microbiota of subjects subjected to nutritional interventions, including the species of Bifidobacteria and *S. mutans* to help understand the relationship between changes in microbial communities and weight control.

The study found a large difference in salivary *S. mutans* and Bifidobacteria percentages according to the body mass index and a positive relation between body fat accumulation and salivary Bifidobacteria count, thus suggesting that saliva specific microbial load may be a marker of excess weight in childhood.

## Data Availability Statement

The raw data supporting the conclusions of this article will be made available by the authors, without undue reservation.

## Ethics Statement

The studies involving human participants were reviewed and approved by Research Ethics Committee of the Piracicaba School of Dentistry (University of Campinas, Brazil; protocol no. 152/2014). Written informed consent to participate in this study was provided by the participants' legal guardian/next of kin.

## Author Contributions

DA, MK, FF, and PC participated in the study design and conception of the study. DA and KS collected the data and biological samples. DA, LS, and MP participated in laboratory analysis. MK and FF supervised the laboratory analysis. PC performed the statistical analysis. DA, TP, CF, and PC wrote the manuscript. All authors reviewed and approved the final version of the manuscript.

## Conflict of Interest

The authors declare that the research was conducted in the absence of any commercial or financial relationships that could be construed as a potential conflict of interest.
