# Supplementary material for: Salivary Microbiological and Gingival Health Status Evaluation of Adolescents With Overweight and Obesity: A Cluster Analysis
Source: Front Pediatr. 2020 Jul 31;8:429. doi: 10.3389/fped.2020.00429 (PMC7411150; doi:10.3389/fped.2020.00429)
Supplement: Supplementary file 1 [file Table_1.DOC]

STROBE Statement—Checklist of items that should be included in reports of ***cross-sectional studies***

***Title: Salivary microbiological evaluation of adolescents with overweight and obesity: a cluster analysis***

|  | Item No | Recommendation | Page/section |
| --- | --- | --- | --- |
| **Title and abstract** | 1 | (*a*) Indicate the study’s design with a commonly used term in the title or the abstract | This information was included in the abstract “cross-sectional relationship” |
|  |  | (*b*) Provide in the abstract an informative and balanced summary of what was done and what was found | Done. |
| Introduction | | |  |
| Background  /rationale | 2 | Explain the scientific background and rationale for the investigation being reported | Introduction section |
| Objectives | 3 | State specific objectives, including any prespecified hypotheses | Aim and hypothesis (last paragraph) |
| Methods | | |  |
| Study design | 4 | Present key elements of study design early in the paper | 1^st^ paragraph: “This work is a cross-sectional analytical study…” |
| Setting | 5 | Describe the setting, locations, and relevant dates, including periods of recruitment, exposure, follow-up, and data collection | Material and Methods, 1^st^ paragraph |
| Participants | 6 | (*a*) Give the eligibility criteria, and the sources and methods of selection of participants | Material and Methods, 1^st^ and 2^nd^ paragraphs |
| Variables | 7 | Clearly define all outcomes, exposures, predictors, potential confounders, and effect modifiers. Give diagnostic criteria, if applicable | Material and Methods section and sub-sections |
| Data sources/ measurement | 8* | For each variable of interest, give sources of data and details of methods of assessment (measurement). Describe comparability of assessment methods if there is more than one group | Material and Methods section and sub-sections |
| Bias | 9 | Describe any efforts to address potential sources of bias | The effort to control for potential sources of bias are summarized in the exclusion criteria, as well in the examination of oral health. |
| Study size | 10 | Explain how the study size was arrived at | Material and Methods, 2nd paragraph (sample size calculation) |
| Quantitative variables | 11 | Explain how quantitative variables were handled in the analyses. If applicable, describe which groupings were chosen and why | Material and Methods section; Physical examination sub-section |
| Statistical methods | 12 | (*a*) Describe all statistical methods, including those used to control for confounding | Material and Methods, Statistical analysis sub-section |
|  |  | (*b*) Describe any methods used to examine subgroups and interactions |  |
|  |  | (*c*) Explain how missing data were addressed | Tables show the exact number of participants enrolled in each comparison. |
|  |  | (*d*) If applicable, describe analytical methods taking account of sampling strategy | NA |
|  |  | (*e*) Describe any sensitivity analyses | NA |
| Results | | |  |
| Participants | 13* | (a) Report numbers of individuals at each stage of study—eg numbers potentially eligible, examined for eligibility, confirmed eligible, included in the study, completing follow-up, and analysed | The number and characteristics of the participants are described in the tables. |
|  |  | (b) Give reasons for non-participation at each stage | NA (participants who did not participate in any examination were excluded) |
|  |  | (c) Consider use of a flow diagram | NA |
| Descriptive data | 14* | (a) Give characteristics of study participants (eg demographic, clinical, social) and information on exposures and potential confounders | Table 1 |
|  |  | (b) Indicate number of participants with missing data for each variable of interest | Table 2 shows the exact number of individuals who were included in the analysis (emphasizing any missing). |
| Outcome data | 15* | Report numbers of outcome events or summary measures | Outcomes were described in the Results section and Figures 1-3. |
| Main results | 16 | (*a*) Give unadjusted estimates and, if applicable, confounder-adjusted estimates and their precision (eg, 95% confidence interval). Make clear which confounders were adjusted for and why they were included | Confounders were considered in the eligibility of the participants (inclusion and exclusion factors) |
|  |  | (*b*) Report category boundaries when continuous variables were categorized | NA (All continuous variables were analysed using raw data) |
|  |  | (*c*) If relevant, consider translating estimates of relative risk into absolute risk for a meaningful time period | NA |
| Other analyses | 17 | Report other analyses done—eg analyses of subgroups and interactions, and sensitivity analyses | The results of interaction analysis are shown in Table 2. |
| Discussion | | |  |
| Key results | 18 | Summarise key results with reference to study objectives | The key results are discussed in the first paragraph. |
| Limitations | 19 | Discuss limitations of the study, taking into account sources of potential bias or imprecision. Discuss both direction and magnitude of any potential bias | The main limitations and strengths of the study were included in the Discussion (last paragraphs), as well the external validity of the study was considered in the text. |
| Interpretation | 20 | Give a cautious overall interpretation of results considering objectives, limitations, multiplicity of analyses, results from similar studies, and other relevant evidence |  |
| Generalisability | 21 | Discuss the generalisability (external validity) of the study results |  |
| Other information | | |  |
| Funding | 22 | Give the source of funding and the role of the funders for the present study and, if applicable, for the original study on which the present article is based | Funding sources were included in the Acknowledgements |
